# The role of social deprivation and depression in dementia risk: findings from the longitudinal survey of health, ageing and retirement in Europe

**DOI:** 10.1017/S2045796023000033

**Published:** 2023-02-14

**Authors:** L. M. Hofbauer, F. S. Rodriguez

**Affiliations:** Research Group Psychosocial Epidemiology and Public Health, German Center for Neurodegenerative Diseases (DZNE), Site Rostock/Greifswald, Ellernholzstr 1-2, 17489 Greifswald, Germany

**Keywords:** Dementia, depression, economic issues, social deprivation, social environment

## Abstract

**Aims:**

Knowledge on the link of individual social deprivation with dementia is incomplete. We thus aimed to see whether an association with dementia risk can be observed using a recently developed Social Deprivation Index (SoDep Index). Further, as deprivation is related to depression, we investigated the role of depression in the association.

**Methods:**

We analysed data of 11 623 Survey of Health, Ageing and Retirement in Europe (SHARE) respondents. Social deprivation status was determined by SoDep Index score. Dementia was determined by self-reported diagnosis. Dementia risk by social deprivation status was estimated using Cox proportional hazard models, including relevant covariates (gender, marriage status, chronic conditions). Depressive symptom status was added in a second step. Further, we completed subgroup analyses by social deprivation status and analysed the relevance of depressive symptoms in dementia risk in each deprivation group. In an additional sensitivity analyses we corrected for mortality and used impaired cognitive testing performance as an alternative outcome.

**Results:**

High (*v*. low) social deprivation status was associated with an increased dementia risk (hazard ratio (HR) = 1.79 [95% CI 1.31–2.45]) in the Cox analysis adjusted for covariates only. Further adjustment for depressive symptom status indicated a largely direct association between social deprivation status and dementia risk. Moreover, compared to not having experienced depressive symptoms in the past or at baseline, those with past (HR = 1.67 [95% CI 1.23–2.25]), baseline (HR = 1.48 [95% CI 1.04–2.10]) or stable depressive symptoms (HR = 2.96 [95% CI 2.12–4.14]) had an increased dementia risk. The association between stable depressive symptom status and dementia risk was in the high social deprivation subgroup particularly pronounced. Sensitivity analyses replicated results.

**Conclusions:**

Results add to a growing body of evidence indicating that a public health approach to dementia prevention must address socioeconomic inequity. Results suggest a largely direct association between social deprivation and dementia risk. Adults who experience high social deprivation appear particularly affected by detrimental effects of depressive symptomatology on dementia risk and need intervention.

## Introduction

As much as one-third of premature deaths seem to be attributable to socioeconomic disadvantage, as data from England show (Lewer *et al*., 2020). Indeed, the role of lacking socioeconomic resources in ill health is widely acknowledged. The construct of socioeconomic status (SES), defined by education, income and/or occupation, has been commonly used to this end. SES has shown a robust association with mental (e.g. Reiss *et al*., 2019), physical (e.g. Kivimäki *et al*., 2020) and cognitive health (e.g. Lyu and Burr, 2016; Marden *et al*., 2017).

The construct of SES has been criticised as too restrictive to reflect the complex reality of deprivation (Salmond *et al*., 2006; Czajka and Denmead, 2008). As a result, studies using multi-domain individual social deprivation indicators have emerged (e.g. Chung *et al*., 2018; Dunlop *et al*., 2022). Multi-domain assessments on the *area* level – such as the Townsend Deprivation Index (Townsend *et al*., 1988) or the Area Deprivation Index (Kind *et al*., 2014) – reflect the degree to which an individual possesses inadequate resources relative to the societal standard. This is particularly relevant to studying older adults, who rely increasingly on resources other than income, such as wealth and health care (O'Reilly, 2002; Galobardes *et al*., 2006; Steptoe and Zaninotto, 2020).

Multi-domain individual social deprivation indicators have demonstrated an association of deprivation with mental and physical health (e.g. Saito *et al*., 2014; Pförtner and Elgar, 2016; Chung *et al*., 2018). However, cognitive outcomes have received less attention. Therefore, our group has recently investigated the association between social deprivation and cognitive function in dementia-free older adults. To this end, we constructed a measure of social deprivation, the Social Deprivation Index (SoDep Index), a brief yet comprehensive index measure of five domains (years of education, income, wealth, health insurance status, lifetime job stability). SoDep Index scores have been found to be related to standardised measurements of cognitive status and decline in 11 101 respondents in the US Health and Retirement Study (HRS; Hofbauer and Rodriguez, 2021a) as well as 51 630 respondents of the Survey of Health and Retirement in Europe (SHARE; Hofbauer and Rodriguez, 2021b).

We expect that this association of individual social deprivation with cognitive function in dementia-free older adults may extend to an association with dementia risk. While this has not been investigated directly, indication is given by previous research linking aspects of social deprivation, such as low wealth (Cadar *et al*., 2018) and low education (Xu *et al*., 2016), to dementia risk. Such an association may be explained by the social causation hypothesis. It postulates that individuals with high social deprivation are more likely to experience chronic stressors, such as financial worry and social defeat (Almeida *et al*., 2005; Hudson, 2005). Evidence from rodent models suggests that chronic stressors induce harmful physiological processes, such as chronic inflammation, including neuroinflammation (Calcia *et al*., 2016). Observational studies in humans appear to support this, with chronic stress showing an association with neuropathology and -degeneration (Peña-Bautista *et al*., 2020).

If chronic stressors are involved in a pathway linking social deprivation and dementia risk, this is likely to be reflected in an indirect effect of social deprivation on dementia risk via depression. This is because social deprivation has repeatedly been shown to be associated with depression (e.g. Fernández-Niño *et al*., 2014; Wickham *et al*., 2014; Cohen-Cline *et al*., 2018; Ye *et al*., 2021), and the association is mediated by chronic stressors (Wickham *et al*., 2014). Depression, in turn, is related to poor cognition (Shaw *et al*., 2022). It is associated with pathological brain changes (Geerlings *et al*., 2013; Belzung *et al*., 2015) and with dementia risk (Livingston *et al*., 2020). Indeed, recently it has been found that depression partially mediates the relationship between *area* social deprivation and cognition (Shaw *et al*., 2022). However, whether this extends to dementia risk remains to be shown. Further, as the previous finding was made on an area level, it would be informative to see whether this can be replicated using a comprehensive *individual*-level social deprivation indicator.

Depression might be particularly detrimental in high social deprivation which could account for a higher dementia risk. Depression has been found to be more persistent in those experiencing socioeconomic disadvantage (Finegan *et al*., 2018) and those living in deprived areas (Finegan *et al*., 2020), compared to those who experience less disadvantage. Factors such as the demoralising effect of living a deprived environment, lack of self-esteem due to upward social comparison, and poor access to health-enhancing goods may be creating a downward spiral (Finegan *et al*., 2020). This downward spiral may further exacerbate depression-related brain changes and ultimately result in less available resources when it comes to resisting dementia.

### Aim

We aim to establish whether social deprivation is a risk factor for dementia, expecting higher social deprivation to be associated with a higher risk of (self-reported) dementia. Further, we inspected the role of depression in the association between social deprivation and dementia risk. We hypothesise that the association between depression and dementia risk will vary with social deprivation status, with a stronger negative effect in those more deprived.

## Method

### Sample

**Fig. 1.**
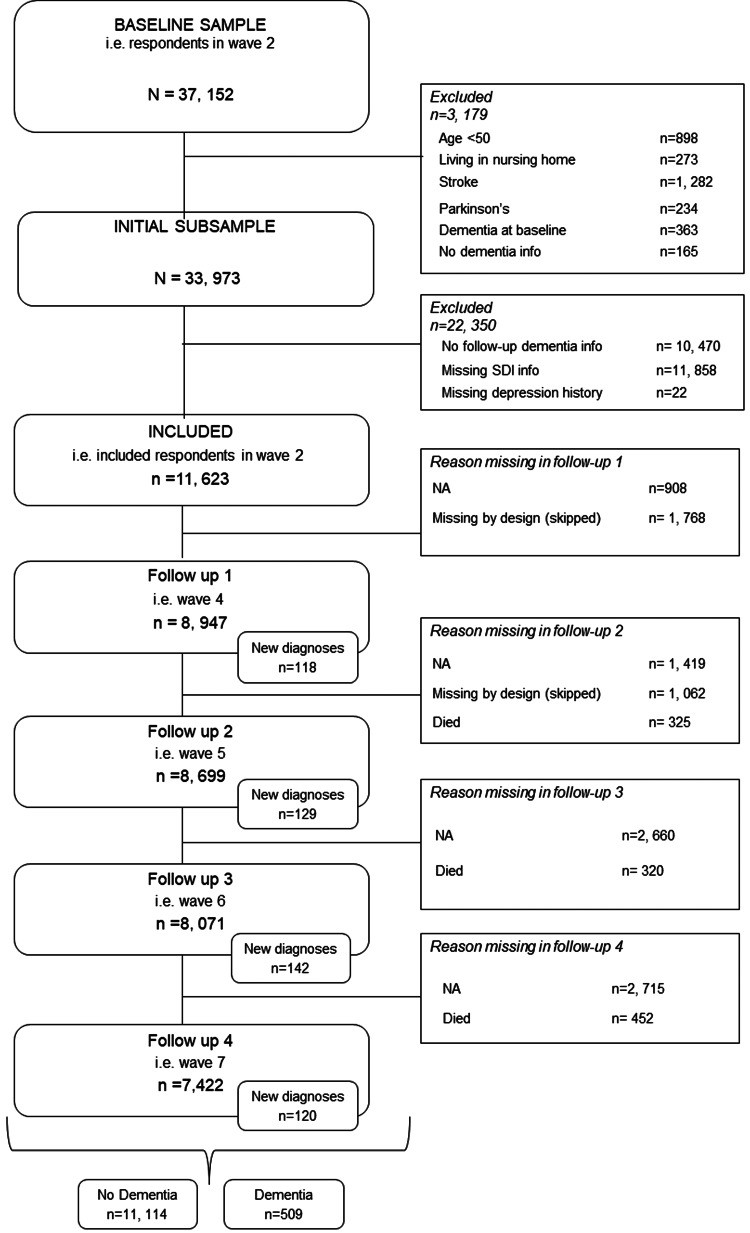
Flow chart showing respondent selection.

The SHARE is a longitudinal cross-country study of Europeans and Israelis 50 years and older. Ethic committees at the University of Mannheim and the Max Planck society have approved this study. All participants provided written informed consent. The first wave of data collection took place in 2004; since then, data collection has been taking place approximately every 2 years. Due to the Covid-19 pandemic, data collection has been limited for wave 8, so that, at the time of writing, the most recent complete data collection was that for wave 7 in 2017. In the present analysis, we included data from waves 2–7, as dementia status was not assessed in wave 1. In wave 3, data collection was restricted to retrospective life history data (SHARELIFE). We include respondents who at baseline (wave 2) reported being dementia-free and having neither a past nor current stroke/cerebral cardiovascular disease nor Parkinson's disease. We further limited inclusion to participants for whom information on covariates, depression symptomatology and social deprivation was available and who were at least 50 years of age and not in institutional care at baseline. Further, we excluded respondents who were never re-interviewed after baseline. The final sample contained 11 623 respondents (see Fig. 1).

### Social Deprivation Index (SoDep Index) Status

The SoDep Index was originally conceptualised in the US HRS sample (Hofbauer and Rodriguez, 2021a) and has since been validated in the SHARE sample (Hofbauer and Rodriguez, 2021b). Domains included are education, income, wealth, job stability and health insurance status. Education is measured in years. Income sums all reported household incomes. Wealth expresses a household's net financial assets less the household's financial liabilities. Income and wealth were adjusted for the household size by dividing by the square root of household size. To determine job stability (i.e. having held a job for 5+ years) we extracted employment history from the retrospective SHARELIFE data. For health insurance status, we recorded whether respondents had any supplementary health insurance beyond basic national insurance.

All domains were reverse coded, so that a greater value indicated greater deprivation. We then multiplied the domains with weights determined by structural equation modelling coefficients during index construction (see Hofbauer and Rodriguez, 2021a). The coefficients are shown in Table 1. Finally, we sub-divided the sample according to SoDep Index Status. Respondents with high SoDep Index scores (⩾80% percentile) were considered to have high SoDep Index Status, low SoDep Index scorers (⩽20% percentile) were considered to have low SoDep Index Status. These percentiles are commonly operationalised as indicating ‘high’ and ‘low’ socioeconomic position (e.g. Krieger *et al*., 2014; Stenberg *et al*., 2019). Accordingly, remaining respondents (21–79% percentile) were identified as having moderate SoDep Index Status.

**Table 1.** SoDep Index domains with corresponding weights based on Hofbauer and Rodriguez 2021a

Index domain^1^	SEM coefficient
Years of education	0.59
Income	0.80
Wealth	0.52
Health insurance status	0.42
Job stability	0.47

SEM, structural equation modelling.

1 Index domains were reverse scored so that a higher value on the index reflects greater deprivation experience.

### Age at self-reported dementia diagnosis

We considered participants who responded in the affirmative to ‘Has a doctor ever told you that you had/Do you currently have: Alzheimer's disease, dementia, organic brain syndrome, senility or any other serious memory impairment’ as having dementia. While this is neither specific, nor a confirmed clinical diagnoses, it is established practice in the handling of SHARE data (e.g. Ferreira *et al*., 2020). Thus, throughout this manuscript, we use ‘dementia’ when referring to self-reported diagnosis. We took the halfway point between the last wave at which respondents reported being dementia-free and the first wave they reported a diagnosis as the time of self-reported diagnosis (see also e.g. Wang *et al*., 2012; Wu *et al*., 2020). We used baseline age and the time to diagnosis to determine age at self-reported diagnosis.

### Age at death

SHARE interviewers record at each wave whether a respondent is alive or has died, confirming this vital status wherever possible with someone in the respondents close social network (Bergmann *et al*., 2019). We took the halfway point between last wave alive and first wave respondents were recorded dead as the time of death to calculate the age at death.

### Depressive symptom status

We used a composite measure reflecting whether respondents had in the past experienced symptoms indicative of depression and/or whether they were experiencing them at baseline. A distinction between past and baseline depression was made given differential effects of early- and late-onset depression on cognitive decline (Jamieson *et al*., 2019; Manning *et al*., 2022). To determine past depressive symptoms, respondents were asked whether there had been a time or times in their life, when they suffered from symptoms of depression which lasted at least 2 weeks. Baseline depressive symptoms were recorded using the EURO-D scale, with scores ⩾6 being deemed indicative of depression at baseline (Tomás *et al*., 2022). Respondents were grouped according to their report of depressive symptoms (1) neither in the past nor at baseline, (2) in the past but not a baseline (‘*past depressive symptoms*’) (3) at baseline but not in the past (‘*baseline depressive symptoms*’), (4) both in the past and at baseline (‘*stable depressive symptoms*’).

### Baseline covariates

Covariates recorded at wave 2 (baseline in this study) were respondents' gender, whether respondents were married/partnered or not (i.e. widowed/divorced/never married) and the number of chronic health conditions respondents reported having been diagnosed with (for the list of conditions considered see online Supplementary Table S1). Covariates were chosen based on evidence showing an association with dementia risk and depressive symptomatology. Being married or partnered has beneficial effects on cognition (Sommerlad *et al*., 2018) and mitigates negative effects of deprivation on mental health (Bierman, 2009). Both risk of dementia (Nebel *et al*., 2018) and depression (Girgus *et al*., 2017) are generally higher in women. Poor overall health is also associated with greater dementia risk (Stephan *et al*., 2021) as well as depression (Chang-Quan *et al*., 2009). Baseline covariates are chosen to minimise the risk of reverse causality (see e.g. VanderWeele *et al*., 2016).

### Analyses

All analyses were conducted using R (Version 4.2.2.) in RStudio (RStudio Team, 2020). We set the significance level *α* < 0.05.

We calculated the mean and standard deviation (s.d.), or the median and interquartile range, of participant demographics. We assessed group differences at baseline between respondents with and without dementia. For continuous variables we employed Mann–Whitney *U* tests based on violations of the homogeneity of variance assumption. Age was the exception to this and compared using a *t* test. For categorical variables, we used *χ*^2^ tests. In addition, we completed a *χ*^2^ tests to determine whether there was a significant association between SoDep Index Status and dementia as well as between SoDep Index Status and Depressive Symptoms Status.

For all survival analyses, time to event was defined as lifetime to event (i.e. age). Given the close link between dementia and age, age has been identified as the appropriate timescale to use in dementia-related analyses (Thiébaut and Bénichou, 2004; Weuve *et al*., 2015). The time at risk was defined as the lifetime between birth and either dementia or censure (death/last contact). For each SoDep Index Status, we calculated the total time at risk and the incidence per 1000 person-years. In addition, we completed Kaplan–Maier analyses to estimate the median age of dementia-free survival by SoDep Index Status, i.e. the age at which the probability of dementia-free survival reached 50%. Further, we estimated the probability of being dementia-free at 70 and 90 years of age.

In a multivariate Cox proportional hazards model (R package: ‘survival’; Therneau, 2021), we included, dementia status (dementia or censored) and age at last follow-up (age at self-reported diagnosis, death, end of participation or end of study) as outcomes. Predictors, entered simultaneously, were SoDep Index Status and baseline covariates. In a second step, we extended this model by adding Depressive Symptom Status as a predictor. This step-wise approach allowed us to first estimate the total variance in dementia risk explained by SoDep Index Status before identifying the variance explained by SoDep Index Status directly. Further, we intended to investigate an interaction between SoDep Index Status with Depressive Symptoms Status. Yet, this would have resulted in a multitude of crossing hazard curves, indicating that this was not appropriate (see online Supplementary Fig. S1). Thus, we repeated the hazard analyses separately for each SoDep Index Status (low, moderate, high) to determine whether the association between depression history and dementia risk would vary depending on SoDep Index status.

To account for the fact that respondents who die may in the future have reported a dementia diagnosis (i.e. the competing risk of death), we completed an additional analysis using a Fine–Gray subdistribution hazard model (R package: ‘cmprsk’; Gray, 2020). The Fine–Gray model included event status (dementia, death or censored) and age at last follow-up as outcomes and SoDep Index Status, baseline covariates and Depressive Symptom Status as predictors. In addition, we completed a sensitivity analysis using cognitive performance scores (see online Supplementary Text S1).

## Results

### Descriptive characteristics

At baseline, respondents in the survey were on average 64.86 (s.d.: 8.63) years old, and 52.16% were female. They were followed for an average 8.70 (s.d.: 2.17) years. A total of 509 respondents (4.38%) reported dementia over the follow-up period. At baseline, those who went on to develop dementia were, on average, older, had undergone fewer years of education, were less likely to be married/partnered, had more chronic conditions, more frequently had experienced past, present or stable depressive symptoms and had lower median incomes and wealth (see online Supplementary Table S2).

SoDep Index Status was significantly associated with dementia (*χ*^2^ (2) = 34.66, *p* < 0.001). The association between SoDep Index Status and Depressive Symptoms was also significant (*χ*^2^ (6) = 163, *p* < 0.001). For percentages see Table 2.

**Table 2.** Sample demographics

	Total	Low SoDep Index Status	Moderate SoDep Index Status	High SoDep Index Status
*N* =	11 623	2325	6973	2325
Gender				
Female	6062 (52.2%)	1082 (46.5%)	3800 (54.5%)	1180 (50.8%)
Male	5561 (47.8%)	1243 (53.5%)	3173 (45.5%)	1145 (49.2%)
Age *mean* (s.d.)	64.86 (8.63)	63.04 (7.80)	65.59 (8.73)	64.51 (8.81)
Age *range*	50–97	50–90	50–97	50–96
Yrs education *mean* (s.d.)	10.92 (4.45)	16.43 (2.60)	10.19 (3.16)	7.62 (4.40)
Yrs education *range*	0–25	10–25	0–25	0–20
Marriage status				
Married/partnered	8779 (75.5%)	1877 (80.7%)	5162 (74%)	1740 (74.8%)
Widowed/divorced/never married	2844 (24.5%)	448 (19.3%)	1811 (26%)	585 (25.2%)
Chronic conditions *mean* (s.d.)	1.43 (1.36)	1.19 (1.18)	1.44 (1.35)	1.65 (1.51)
Chronic conditions *range*	0–9	0–7	0–9	0–9
Depressive symptom status				
No past/baseline depressive symptoms	9847 (84.7%)	2044 (87.9%)	5993 (85.9%)	1810 (77.8%)
Past depressive symptoms	972 (8.4%)	192 (8.3%)	551 (7.9%)	229 (9.8%)
Baseline depressive symptoms	410 (3.5%)	50 (2.2%)	223 (3.2%)	137 (5.95%)
Stable depressive symptoms	394 (3.4%)	39 (1.7%)	206 (3%)	149 (6.4%)
Income *median* (*IQR*)	23 064.11 (25 728.62)	41 275.20 (34 710.45)	21 965.32 (20 226.10)	12 277.86 (16 852.60)
Income *Range*	0–1 218 168	868.621–1 218 168	0–1 021 803.63	0–305 751.91
Wealth *Median* (*IQR*)	15 333.49 (65 210.59)	100 671.14 (172 181.08)	17 836.92 (49 000)	0 (7000)
Wealth *Range*	−505 782.32–1 789 785.69	0–1 789 785.69	−19 054.77–1 019 355.83	−505 782.32–1 444 961.44
Health Insurance Status				
Basic	5028 (43.3%)	838 (36%)	3910 (56.1%)	1847 (79.4%)
Supplementary	65 995 (56.7%)	1487 (64%)	3063 (43.9%)	487 (20.6%)
Ever held a job for 5 + years				
Yes	11 215 (96.5%)	2288 (98.4%)	6705 (96.2%)	2222 (95.65)
No	408 (3.5%)	37 (1.6%)	268 (3.8%)	103 (4.4%)
*Follow-up variables*				
Self-reported dementia diagnosis				
No	11 114 (95.6%)	2269 (97.6%)	6657 (95.5%)	2188 (94.1%)
Yes	509 (4.4%)	56 (2.4%)	316 (4.5%)	137 (5.9%)
Age diagnosis *mean* (s.d.)	79.78 (8.02)	78.73 (8.09)	80.20 (7.12)	79.23 (8.65)
Age diagnosis *range*	54–102	60–98	56–102	54–99
Age at death *mean* (s.d.)	81.10 (9.29)	78.72 (9.41)	81.68 (9.08)	82.10 (9.56)
Age at death *range*	56–104	57–100	58–104	56–101
Age at end of follow-up *mean* (s.d.)	73.57 (8.52)	71.83 (7.84)	74.21 (8.63)	73.38 (8.61)
Age to end of follow-up *range*	54–103	54–100	54–103	54–101

EURO-D, European Depressive Symptoms Scale, IQR, interquartile range; *N*, total number; NA, not applicable; s.d., standard deviations, Yrs, years.

### Dementia-free survival by SoDep index status

The total person-years at risk and incidence rates per 1000 person-years were the following: 166 997 person-years (incidence: 0.34) for low SoDep Index Status, 517 446 person-years (incidence: 0.61) for moderate SoDep Index Status and 170 614 person-years (incidence: 0.80) for high SoDep Index Status. Results from the Kaplan–Mayer estimation showed that the median dementia-free age reached was 102 in the moderate SoDep Index Status group and 99 in the high SoDep Index Status group. For those with low SoDep Index Status, the median was not yet reached, i.e. the probability of dementia-free survival did not drop to 50% in the observed data for this group. The probability of being dementia-free at age 70 was 99.41% (95% CI 99.04–99.78) for low, 99.40% (95% CI 99.20–99.61) for moderate and 98.69% (95% CI 98.16–99.22) for high SoDep Index Status. At age 90, the probabilities were 82.86% (95% CI 77.25–88.86), 80.85% (95% CI 78.42–83.36) and 72.73% (95% CI 67.31–78.58), respectively.

### SoDep index status, depressive symptom status and dementia

**Fig. 2.**
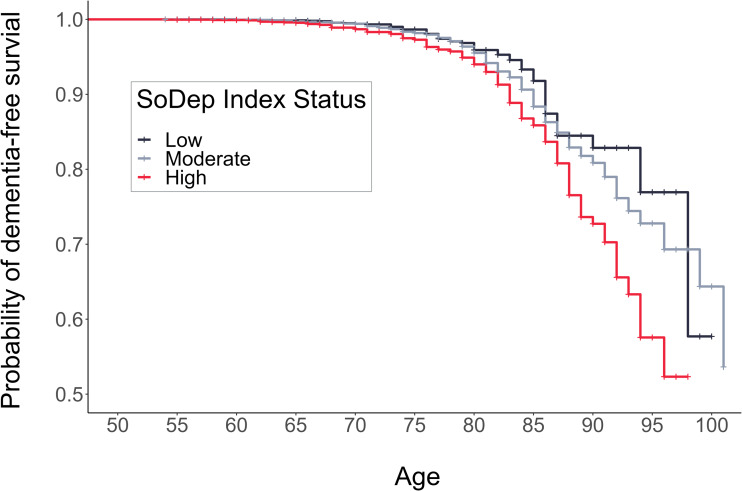
Cumulative Incidence Function showing risk of a dementia diagnosis by age and SoDep Index Status.

Cox regression results showed high SoDep Index Status to be associated with increased dementia risk (HR = 1.79, [95% CI 1.31–2.45], *p* < 0.001), compared to low SoDep Index Status (see Fig. 2). After adding Depressive Symptom Status as a predictor, the HR for high SoDep Index Status changed to 1.63 ([95% CI 1.19–2.24], *p* = 0.002). In conjunction, these findings indicate that high (*v*. low) SoDep status was associated with a 79% increase in dementia risk and that Depressive Symptom Status accounted for 16% of this difference in risk. In addition, compared to not having past or baseline depressive symptoms, each Depressive Symptom Status was associated with an increased dementia risk (past depressive symptoms: HR = 1.66, [95% CI 1.23–2.24], *p* *<* 0.001; baseline depressive symptoms: HR = 1.48, [95% CI 1.04–2.10], *p* = 0.030; stable depressive symptoms: 2.96, [95% CI 2.12–4.14], *p* < 0.001). For full model output see Table 3. These findings held up to adjustment for mortality (see online Supplementary Table S3) and were replicated for cognitive performance (see online Supplementary Text S1).

**Table 3.** Cox regression results for the full sample

	Model without Depressive Symptom Status			Model with Depressive Symptom Status		
Variable	HR (95% CI)	*p* Value	s.e.	HR (95% CI)	*p* Value	s.e.
SDI						
Low	REF			REF		
Moderate	1.23 (0.92–1.64)	*0*.*156*	0.13	1.21 (0.91–1.61)	*0*.*190*	0.18
High	1.79 (1.31–2.45)	***<0***.***001***	0.29	1.63 (1.19–2.24)	***0***.***002***	0.26
Depressive Symptom Status						
No past/baseline depressive symptoms				REF		
Past depressive symptoms				1.66 (1.23–2.24)	***<0***.***001***	0.26
Baseline depressive symptoms				1.48 (1.04–2.10)	***0***.***030***	0.26
Stable depressive symptoms				2.96 (2.12–4.14)	***<0***.***001***	0.51
Gender						
Female	REF			REF		
Male	0.78 (0.64–0.94)	***0***.***008***	0.07	0.83 (0.69–1.01)	*0*.*057*	0.08
Marriage status						
Married/partnered	REF			REF		
Not married/partnered	0.66 (0.54–0.81)	***<0***.***001***	0.07	0.66 (0.54–0.80)	***<0***.***001***	0.07
Chronic conditions	1.00 (0.94–1.06)	*0*.*965*	0.03	0.96 (0.91–1.02)	*0*.*226*	0.03
Wald-test	36.95			88.02		
*p* Value	*<0.001*			*<0*.*001*		
df	5			8		
BIC	7879			7852		

BIC, Bayesian information criterion; CI, confidence interval; df, degrees of freedom; HR, hazard ratio; REF, indicates the reference level of a variable; s.e., standard error.

### SoDep index status subgroup analyses

In subgroup analyses, stable depressive symptoms, compared to no past or baseline depressive symptoms, were associated with higher dementia risk in the moderate (HR = 2.11, [95% CI 1.26–3.54], *p* *=* 0.005) and high (HR = 4.37, [95% CI 2.65–7.22], *p* *<* 0.001) SoDep Index Status group. That is, the dementia risk was more than doubled in those with moderate SoDep Status and stable depressive symptoms compared to those with moderate SoDep Index Status and no past or baseline depressive symptoms. For those with high SoDep Index Status and stable depressive symptoms the dementia risk was more than quadrupled compared to those with high SoDep Index Status and no past or baseline depressive symptoms. In addition, for those with moderate SoDep Index Status past depressive symptoms were associated with increased risk (HR = 1.70, [95% CI 1.16–2.49], *p* *=* 0.007) compared to no past or baseline depressive symptoms. For the low SoDep Index Status group, none of the predictors were significant (see Table 4).

**Table 4.** Cox regression results for SoDep Index Status subgroups

	Social Deprivation Status								
	Low SoDep Index Status			Moderate SoDep Index Status			High SoDep Index Status		
Variable	HR (95% CI)	*p* Value	s.e.	HR (95% CI)	*p* Value	s.e.	HR (95% CI)	*p* Value	s.e.
Depressive Symptom Status									
No past/baseline depressive symptoms	REF			REF			REF		
Past depressive symptoms	1.88 (0.80–4.41)	*0*.*150*	0.82	1.70 (1.16–2.49)	***0***.***007***	0.33	1.64 (0.91–2.96)	*0*.*100*	0.49
Baseline depressive symptoms	1.83 (0.42–7.93)	*0*.*421*	1.37	1.45 (0.88–2.40)	*0*.*146*	0.37	1.48 (0.86–2.53)	*0*.*150*	0.40
Stable depressive symptoms	1.79 (0.24–13.42)	*0*.*572*	1.84	2.11 (1.26–3.54)	***0***.***005***	0.56	4.37 (2.65–7.22)	***<0***.***001***	1.12
Gender									
Female	REF			REF			REF		
Male	0.60 (0.36–1.08)	*0*.*064*	0.17	0.84 (0.67–1.07)	*0*.*164*	0.10	0.99 (0.67, 1.45)	*0*.*940*	0.20
Marriage status									
Married/partnered	REF			REF			REF		
Not married/partnered	0.64 (0.33–1.23)	*0*.*182*	0.22	0.59 (0.46–0.76)	***<0***.***001***	0.08	0.88 (0.60, 1.30)	*0*.*520*	0.17
Chronic conditions	0.92 (0.73–1.11)	*0*.*433*	0.10	0.95 (0.88–1.03)	*0*.*183*	0.04	1.00 (0.89, 1.11)	*0*.*960*	0.06
Wald-test	6.82			30.9			41		
*p* Value	*0.30*			*<0*.*001*			*<0*.*001*		
df	6			6			6		
BIC	683			4609			1681		

BIC, Bayesian information criterion; CI, confidence interval; df, degrees of freedom; EURO-D, European Depressive Symptoms Scale; HR, hazard ratio; REF, indicates the reference level of a variable; s.e., standard error.

## Discussion

In this paper, we aimed to investigate the association between individual social deprivation status, as measured by the SoDep Index, and dementia risk, as determined using self-reported dementia diagnosis. Compared to low SoDep Index Status, high SoDep Index Status was associated with a 79% increase in dementia risk. We further investigate the role of depression in this association. Depressive Symptom Status accounted for 16% of the above reported increase. The subgroup analyses revealed that experiencing depressive symptoms both in the past and at baseline, compared to not experiencing any, was associated with an increased dementia risk in those with high or moderate SoDep Index Status but not those with low SoDep Index Status. Past depressive symptoms were associated with increased dementia risk in those with moderate SoDep Index Status. Results held up to mortality correction and were replicated in an analysis impaired cognitive test performance as the outcome.

Thus, our results show that the previously documented association found between individual social deprivation and cognition (Bongue *et al*., 2016; Hofbauer and Rodriguez, 2021a, 2021b) expands to dementia risk. This is in line with the conceptualisation of cognitive ageing as a continuum that ranges from unimpaired to pathologically impaired function, with a shared underlying process (Brayne and Calloway, 1988). Only a relatively small fraction of the variance in dementia risk was explained by depressive symptomatology. This is contrary to expectation that depression could be the pathological manifestation of high social deprivation leading to dementia (Calcia *et al*., 2016; Peña-Bautista *et al*., 2020; Shaw *et al*., 2022). Other pathways not considered in this work likely play a larger role in the association. One prominent candidate is lifestyle. Individuals with restricted access to resources may be less able to conform to a lifestyle that supports healthy cognition. Indeed, recent research has demonstrated that wealth is associated with a less brain-healthy lifestyle, which in turn mediates around half of the wealth-based dementia risk difference (Deckers *et al*., 2019). Future research may clarify whether this can be replicated using a more comprehensive deprivation assessment.

In the subgroup analyses, we found a particularly strong association between stable depressive symptoms and dementia risk in the high SoDep Index group. Social deprivation may result in low cognitive reserve and thus induce greater vulnerability to depression (Barnett *et al*., 2006), with stable depressive symptoms lowering resources to the degree that vulnerability to dementia is exacerbated. However, subgroup analysis may have been underpowered and confidence intervals suggest substantial uncertainty, so that results need to be interpreted with caution. Moreover, being collected in older adults, the data used in this analysis cannot be used to make any causal claims. Reverse causality (i.e. that depression leads to social deprivation) can be argued for, e.g. because depression can lead to job-loss (Lerner *et al*., 2004). Yet, this is unlikely to fully account for the effect, given that socioeconomic disadvantage in childhood is associated with later depression (Angelini *et al*., 2018).

In conjunction with our previous findings, the present results suggest a consistent association of SoDep Index scores with cognitive health outcomes in older adults. It may be developed into a brief assessment tool to identify individuals at high risk. Unlike standard SES measures, the index was constructed and validated in large, older adult samples, thus answering long-standing calls for assessments specific to this group (e.g. Salmond *et al*., 2006; Czajka and Denmead, 2008).

These findings have practical implications. Clinical practitioners should be aware that the association between depressive symptoms and dementia risk varies by social deprivation status, with those with high social deprivation appearing particularly susceptible to detrimental effects. Interventions to individuals with double risk-status should be prioritised. Ultimately, however, individual-level intervention must be combined with population-level prevention (Walsh *et al*., 2022). That is, policy makers must realise the health-threat that social deprivation poses and address inequality. Basic income appears one plausible way to do so, with economic models confirming macroeconomic feasibility (Gibson *et al*., 2020; Luduvice, 2021) and initial trials showing that such an intervention can indeed be successful in boosting health of recipients (Painter, 2016; Ruckert *et al*., 2017; Gibson *et al*., 2020).

Strengths of this study include that we were able to draw on the large, representative sample of the SHARE. The rich data allowed for comprehensive assessment of both social deprivation and depression as risk factors for dementia. Nonetheless, there are some limitations. First, neither dementia nor depressive symptomatology was clinically confirmed. This limits the reliability of results. Therefore, the findings may be biased by insight into symptoms. In both cases, stigma may influence reporting. Second, by excluding those who reported a dementia diagnosis at baseline, we likely excluded those who were particularly exposed to risk factors for dementia, including social deprivation and depressive symptoms. Thus, our findings may somewhat underestimate the associations. Third, covariates were assessed at baseline. They thus cannot reflect any changes that might relate to dementia risk. For instance, if overall health decreased rapidly during follow-up this may account for some of the variance in dementia risk.

In conclusion, social deprivation appears to be an important risk factor for dementia, even after control for other known risk factors and mortality. Depressive symptoms are a particular concern in those who are experiencing moderate or high levels of social deprivation. Thus, physicians should offer help to any patients that present with depressive symptomatology and experience some degree of socioeconomic strain. Greater equity in society is ultimately the only way to address these dementia risk factors on the population level.

## Data Availability

For this study, we make use of the Survey of Health, Ageing and Retirement in Europe (SHARE) data. SHARE data are available to interested researches who register under http://www.share-project.org.

## Supplementary material

For supplementary material accompanying this paper visit http://doi.org/10.1017/S2045796023000033. click here to view supplementary material

## References

[ref1] Almeida DM, Neupert SD, Banks SR and Serido J (2005) Do daily stress processes account for socioeconomic health disparities? The Journals of Gerontology: Series B 60, 34–39. 10.1093/geronb/60.special_issue_2.s34 16251588

[ref2] Angelini V, Howdon DDH and Mierau JO (2018) Childhood socioeconomic status and late-adulthood mental health: results from the survey on health, ageing and retirement in Europe. The Journals of Gerontology: Series B 74, 95–104. 10.1093/geronb/gby028 PMC6941210 29566242

[ref3] Barnett J, Salmond C, Jones P and Sahakian B (2006) Cognitive reserve in neuropsychiatry. Psychological Medicine 36, 1053–1064. 16854246 10.1017/S0033291706007501

[ref4] Belzung C, Willner P and Philippot P (2015) Depression: from psychopathology to pathophysiology. Current Opinion in Neurobiology 30, 24–30. 25218233 10.1016/j.conb.2014.08.013

[ref5] Bergmann M, Kneip T, De Luca G and Scherpenzeel A (2019) *Survey participation in the Survey of Health, Ageing and Retirement in Europe* (*SHARE*), *Wave* 1–7. Working Paper Series 41-2019. Munich Center for the Economics of Aging (MEA): Munich.

[ref6] Bierman A (2009) Marital status as contingency for the effects of neighborhood disorder on older adults’ mental health. The Journals of Gerontology: Series B 64, 425–434. 10.1093/geronb/gbp010 PMC2905133 19251881

[ref7] Bongue B, Colvez A, Amsallem E, Gerbaud L and Sass C (2016) Assessment of health inequalities among older people using the EPICES score: a composite index of social deprivation. The Journal of Frailty & Aging 5, 168–173. 29240316

[ref8] Brayne C and Calloway P (1988) Normal ageing, impaired cognitive function, and senile dementia of the Alzheimer's type: a continuum? The Lancet 331, 1265–1267. 10.1016/s0140-6736(88)92081-8 2897526

[ref9] Cadar D, Lassale C, Davies H, Llewellyn DJ, Batty GD and Steptoe A (2018) Individual and area-based socioeconomic factors associated with dementia incidence in England: evidence from a 12-year follow-up in the English longitudinal study of ageing. JAMA Psychiatry 75, 723–732. 29799983 10.1001/jamapsychiatry.2018.1012 PMC6145673

[ref10] Calcia MA, Bonsall DR, Bloomfield PS, Selvaraj S, Barichello T and Howes OD (2016) Stress and neuroinflammation: a systematic review of the effects of stress on microglia and the implications for mental illness. Psychopharmacology 233, 1637–1650. 26847047 10.1007/s00213-016-4218-9 PMC4828495

[ref11] Chang-Quan H, Xue-Mei Z, Bi-Rong D, Zhen-Chan L, Ji-Rong Y and Qing-Xiu L (2009) Health status and risk for depression among the elderly: a meta-analysis of published literature. Age and Ageing 39, 23–30. 19903775 10.1093/ageing/afp187

[ref12] Chung RY, Chung GK, Gordon D, Wong SY, Chan D, Lau MK, Tang VM and Wong H (2018) Deprivation is associated with worse physical and mental health beyond income poverty: a population-based household survey among Chinese adults. Quality of Life Research 27, 2127–2135. 29761348 10.1007/s11136-018-1863-y

[ref13] Cohen-Cline H, Beresford SAA, Barrington WE, Matsueda RL, Wakefield J and Duncan GE (2018) Associations between neighbourhood characteristics and depression: a twin study. Journal of Epidemiology and Community Health 72, 202–207. 29273630 10.1136/jech-2017-209453 PMC6007871

[ref14] Czajka JL and Denmead G (2008) Income Data for Policy Analysis: A Comparative Assessment of Eight Surveys. Washington: Mathematica Policy Research, Inc.

[ref15] Deckers K, Cadar D, van Boxtel MPJ, Verhey FRJ, Steptoe A and Köhler S (2019) Modifiable risk factors explain socioeconomic inequalities in dementia risk: evidence from a population-based prospective cohort study. Journal of Alzheimer's Disease 71, 549–557. 10.3233/JAD-190541 PMC6839472 31424404

[ref16] Dunlop BW, Rakofsky JJ, Mischoulon D, Mayberg HS, Kinkead B, Nierenberg AA, Ziegler TR, Fava M and Rapaport MH (2022) The United States index of socioeconomic deprivation for individuals (USiDep). Personalized Medicine in Psychiatry 31, 100091. 35937504 10.1016/j.pmip.2022.100091 PMC9355266

[ref17] Fernández-Niño JA, Manrique-Espinoza BS, Bojorquez-Chapela I and Salinas-Rodríguez A (2014) Income inequality, socioeconomic deprivation and depressive symptoms among older adults in Mexico. PLoS ONE 9, e108127. 25250620 10.1371/journal.pone.0108127 PMC4176015

[ref18] Ferreira RG, Brandão MP and Cardoso MF (2020) An update of the profile of older adults with dementia in Europe: findings from SHARE. Aging & Mental Health 24, 374–381. 30588821 10.1080/13607863.2018.1531385

[ref19] Finegan M, Firth N, Wojnarowski C and Delgadillo J (2018) Associations between socioeconomic status and psychological therapy outcomes: a systematic review and meta-analysis. Depression and Anxiety 35, 560–573. 29697880 10.1002/da.22765

[ref20] Finegan M, Firth N and Delgadillo J (2020) Adverse impact of neighbourhood socioeconomic deprivation on psychological treatment outcomes: the role of area-level income and crime. Psychotherapy Research 30, 546–554. 31366303 10.1080/10503307.2019.1649500

[ref21] Galobardes B, Shaw M, Lawlor DA, Lynch JW and Smith GD (2006) Indicators of socioeconomic position (part 1). Journal of Epidemiology & Community Health 60, 7–12. 10.1136/jech.2004.023531 PMC2465546 16361448

[ref22] Geerlings MI, Sigurdsson S, Eiriksdottir G, Garcia ME, Harris TB, Sigurdsson T, Gudnason V and Launer LJ (2013) Associations of current and remitted major depressive disorder with brain atrophy: the AGES-Reykjavik Study. Psychological Medicine 43, 317–328. 22647536 10.1017/S0033291712001110 PMC4244840

[ref23] Gibson M, Hearty W and Craig P (2020) The public health effects of interventions similar to basic income: a scoping review. The Lancet Public Health 5, e165–e176. 32113520 10.1016/S2468-2667(20)30005-0 PMC7208547

[ref24] Girgus JS, Yang K and Ferri CV (2017) The gender difference in depression: are elderly women at greater risk for depression than elderly men? Geriatrics 2, 35. 31011045 10.3390/geriatrics2040035 PMC6371140

[ref25] Gray B (2020) cmprsk: Subdistribution Analysis of Competing Risks. R package version 2.2–10.

[ref26] Hofbauer LM and Rodriguez FS (2021a) Association of social deprivation with cognitive status and decline in older adults. International Journal of Geriatric Psychiatry 36, 1085–1094. 33860548 10.1002/gps.5555

[ref27] Hofbauer LM and Rodriguez FS (2021b) Validation of a social deprivation index and association with cognitive function and decline in older adults. International Psychogeriatrics 33, 1309–1320. 34494514 10.1017/S1041610221000995

[ref28] Hudson CG (2005) Socioeconomic status and mental illness: tests of the social causation and selection hypotheses. American Journal of Orthopsychiatry 75, 3–18. 15709846 10.1037/0002-9432.75.1.3

[ref29] Jamieson A, Goodwill AM, Termine M, Campbell S and Szoeke C (2019) Depression related cerebral pathology and its relationship with cognitive functioning: a systematic review. Journal of Affective Disorders 250, 410–418. 30878653 10.1016/j.jad.2019.03.042

[ref30] Kind AJ, Jencks S, Brock J, Yu M, Bartels C, Ehlenbach W, Greenberg C and Smith M (2014) Neighborhood socioeconomic disadvantage and 30-day rehospitalization: a retrospective cohort study. Annals of Internal Medicine 161, 765–774. 25437404 10.7326/M13-2946 PMC4251560

[ref31] Kivimäki M, Batty GD, Pentti J, Shipley MJ, Sipilä PN, Nyberg ST, Suominen SB, Oksanen T, Stenholm S, Virtanen M, Marmot MG, Singh-Manoux A, Brunner EJ, Lindbohm JV, Ferrie JE and Vahtera J (2020) Association between socioeconomic status and the development of mental and physical health conditions in adulthood: a multi-cohort study. The Lancet Public Health 5, e140–e149. 32007134 10.1016/S2468-2667(19)30248-8

[ref32] Krieger N, Kosheleva A, Waterman PD, Chen JT, Beckfield J and Kiang MV (2014) 50-year Trends in US socioeconomic inequalities in health: US-born Black and White Americans, 1959–2008. International Journal of Epidemiology 43, 1294–1313. 24639440 10.1093/ije/dyu047 PMC4121555

[ref33] Lerner D, Adler DA, Chang H, Lapitsky L, Hood MY, Perissinotto C, Reed J, McLaughlin TJ, Berndt ER and Rogers WH (2004) Unemployment, job retention, and productivity loss among employees with depression. Psychiatric Services 55, 1371–1378. 15572564 10.1176/appi.ps.55.12.1371 PMC4283817

[ref34] Lewer D, Jayatunga W, Aldridge RW, Edge C, Marmot M, Story A and Hayward A (2020) Premature mortality attributable to socioeconomic inequality in England between 2003 and 2018: an observational study. The Lancet Public Health 5, e33–e41. 31813773 10.1016/S2468-2667(19)30219-1 PMC7098478

[ref35] Livingston G, Huntley J, Sommerlad A, Ames D, Ballard C, Banerjee S, Brayne C, Burns A, Cohen-Mansfield J, Cooper C, Costafreda SG, Dias A, Fox N, Gitlin LN, Howard R, Kales HC, Kivimäki M, Larson EB, Ogunniyi A, Orgeta V, Ritchie K, Rockwood K, Sampson EL, Samus Q, Schneider LS, Selbæk G, Teri L and Mukadam N (2020) Dementia prevention, intervention, and care: 2020 report of the Lancet Commission. The Lancet 396, 413–446. 10.1016/S0140-6736(20)30367-6 PMC7392084 32738937

[ref36] Luduvice AVD (2021) The macroeconomic effects of universal basic income programs. Working paper 21–21. Federal Reserve Bank of Cleveland. 10.26509/frbc-wp-202121.

[ref37] Lyu J and Burr JA (2016) Socioeconomic status across the life course and cognitive function among older adults: an examination of the latency, pathways, and accumulation hypotheses. Journal of Aging and Health 28, 40–67. 26006338 10.1177/0898264315585504

[ref38] Manning KJ, Wu R, McQuoid DR, Steffens DC and Potter GG (2022) Reliable cognitive decline in late-life major depression. Archives of Clinical Neuropsychology, acac083. [Epub ahead of print]. 10.1093/arclin/acac083. PMC9940117 36302229

[ref39] Marden JR, Tchetgen Tchetgen EJ, Kawachi I and Glymour MM (2017) Contribution of socioeconomic status at 3 life-course periods to late-life memory function and decline: early and late predictors of dementia risk. American Journal of Epidemiology 186, 805–814. 28541410 10.1093/aje/kwx155 PMC5859987

[ref40] Nebel RA, Aggarwal NT, Barnes LL, Gallagher A, Goldstein JM, Kantarci K, Mallampalli MP, Mormino EC, Scott L, Yu WH, Maki PM and Mielke MM (2018) Understanding the impact of sex and gender in Alzheimer's disease: a call to action. Alzheimer's & Dementia 14, 1171–1183. 10.1016/j.jalz.2018.04.008 PMC6400070 29907423

[ref41] O'Reilly D (2002) Standard indicators of deprivation: do they disadvantage older people? Age and Ageing 31, 197–202. 12006309 10.1093/ageing/31.3.197

[ref42] Painter A (2016) A universal basic income: the answer to poverty, insecurity, and health inequality? BMJ 355, i6473. 27956433 10.1136/bmj.i6473

[ref43] Peña-Bautista C, Casas-Fernández E, Vento M, Baquero M and Cháfer-Pericás C (2020) Stress and neurodegeneration. Clinica Chimica Acta 503, 163–168. 10.1016/j.cca.2020.01.019 31987795

[ref44] Pförtner TK and Elgar FJ (2016) Widening inequalities in self-rated health by material deprivation? A trend analysis between 2001 and 2011 in Germany. Journal of Epidemiology and Community Health 70, 82–89. 26294770 10.1136/jech-2015-205948

[ref45] Reiss F, Meyrose AK, Otto C, Lampert T, Klasen F and Ravens-Sieberer U (2019) Socioeconomic status, stressful life situations and mental health problems in children and adolescents: results of the German BELLA cohort-study. PLoS ONE 14, e0213700. 30865713 10.1371/journal.pone.0213700 PMC6415852

[ref46] Rstudio Team (2020) RStudio: Integrated Development for R. Boston: PBC.

[ref47] Ruckert A, Huynh C and Labonté R (2017) Reducing health inequities: is universal basic income the way forward? Journal of Public Health 40, 3–7. 10.1093/pubmed/fdx006 28158715

[ref48] Saito M, Kondo K, Kondo N, Abe A, Ojima T, Suzuki K and JAGES group (2014). Relative deprivation, poverty, and subjective health: JAGES cross-sectional study. PLoS ONE 9, e111169. 25350284 10.1371/journal.pone.0111169 PMC4211701

[ref49] Salmond C, Crampton P, King P and Waldegrave C (2006) NZiDep: a New Zealand index of socioeconomic deprivation for individuals. Social Science & Medicine 62, 1474–1485. 16154674 10.1016/j.socscimed.2005.08.008

[ref50] Shaw S, Jana A and Kundu S (2022) An analytical pathway of consumption expenditure with neighborhood deprivation and depression on cognition health among elderly in India: a moderated mediation approach. Journal of Affective Disorders 308, 249–258. 35429519 10.1016/j.jad.2022.04.060

[ref51] Sommerlad A, Ruegger J, Singh-Manoux A, Lewis G and Livingston G (2018) Marriage and risk of dementia: systematic review and meta-analysis of observational studies. Journal of Neurology, Neurosurgery & Psychiatry 89, 231–238. 29183957 10.1136/jnnp-2017-316274 PMC5869449

[ref52] Stenberg E, Persson C, Näslund E, Ottosson J, Sundbom M, Szabo E and Näslund I (2019) The impact of socioeconomic factors on the early postoperative complication rate after laparoscopic gastric bypass surgery: a register-based cohort study. Surgery for Obesity and Related Disorders 15, 575–581. 10.1016/j.soard.2019.01.025 30826242

[ref53] Stephan Y, Sutin AR, Luchetti M, Aschwanden D and Terracciano A (2021) Self-rated health and incident dementia over two decades: replication across two cohorts. Journal of Psychiatric Research 143, 462–466. 34311955 10.1016/j.jpsychires.2021.06.036

[ref54] Steptoe A and Zaninotto P (2020) Lower socioeconomic status and the acceleration of aging: an outcome-wide analysis. Proceedings of the National Academy of Sciences 117, 14911–14917. 10.1073/pnas.1915741117 PMC7334539 32541023

[ref55] Therneau T (2021) A package for survival analysis in R. R package version 3.2–13.

[ref56] Thiébaut ACM and Bénichou J (2004) Choice of time-scale in Cox's model analysis of epidemiologic cohort data: a simulation study. Statistics in Medicine 23, 3803–3820. 15580597 10.1002/sim.2098

[ref57] Tomás JM, Torres Z, Oliver A, Enrique S and Fernández I (2022) Psychometric properties of the EURO-D scale of depressive symptomatology: evidence from SHARE wave 8. Journal of Affective Disorders 313, 49–55. 35772626 10.1016/j.jad.2022.06.079

[ref58] Townsend P, Phillimore P and Beattie A (1988) Health and Deprivation: Inequality and the North. London: Croom Helm.

[ref59] VanderWeele TJ, Jackson JW and Li S (2016) Causal inference and longitudinal data: a case study of religion and mental health. Social Psychiatry and Psychiatric Epidemiology 51, 1457–1466. 27631394 10.1007/s00127-016-1281-9

[ref60] Walsh S, Govia I, Wallace L, Richard E, Peters R, Anstey KJ and Brayne C (2022) A whole-population approach is required for dementia risk reduction. The Lancet Healthy Longevity 3, e6–e8. 36098322 10.1016/S2666-7568(21)00301-9

[ref61] Wang HX, Wahlberg M, Karp A, Winblad B and Fratiglioni L (2012) Psychosocial stress at work is associated with increased dementia risk in late life. Alzheimer's & Dementia 8, 114–120. 10.1016/j.jalz.2011.03.001 22404853

[ref62] Weuve J, Proust-Lima C, Power MC, Gross AL, Hofer SM, Thiébaut R, Chêne G, Glymour MM, Dufouil C and MELODEM Initiative (2015) Guidelines for reporting methodological challenges and evaluating potential bias in dementia research. Alzheimer's & Dementia 11, 1098–1109. 10.1016/j.jalz.2015.06.1885 PMC4655106 26397878

[ref63] Wickham S, Taylor P, Shevlin M and Bentall RP (2014) The impact of social deprivation on paranoia, hallucinations, mania and depression: the role of discrimination social support, stress and trust. PLoS ONE 9, e105140. 25162703 10.1371/journal.pone.0105140 PMC4146475

[ref64] Wu JJ, Wang HX, Yao W, Yan Z and Pei JJ (2020) Late-life depression and the risk of dementia in 14 countries: a 10-year follow-up study from the Survey of Health, Ageing and Retirement in Europe. Journal of Affective Disorders 274, 671–677. 32664001 10.1016/j.jad.2020.05.059

[ref65] Xu W, Tan L, Wang HF, Tan MS, Tan L, Li JQ, Zhao QF and Yu JT (2016) Education and risk of dementia: dose-response meta-analysis of prospective cohort studies. Molecular Neurobiology 53, 3113–3123. 25983035 10.1007/s12035-015-9211-5

[ref66] Ye J, Wen Y, Sun X, Chu X, Li P, Cheng B, Cheng S, Liu L, Zhang L, Ma M, Qi X, Liang C, Kafle OP, Jia Y, Wu C, Wang S, Wang X, Ning Y, Sun S and Zhang F (2021) Socioeconomic deprivation index is associated with psychiatric disorders: an observational and genome-wide gene-by-environment interaction analysis in the UK Biobank Cohort. Biological Psychiatry 89, 888–895. 33500177 10.1016/j.biopsych.2020.11.019

