# Supplementary material for: The role of social deprivation and depression in dementia risk: findings from the longitudinal survey of health, ageing and retirement in Europe
Source: Epidemiol Psychiatr Sci. 2023 Feb 14;32:e10. doi: 10.1017/S2045796023000033 (PMC9971857; doi:10.1017/S2045796023000033)
Supplement: Supplementary file 1 [file S2045796023000033sup001.docx]

Supplementary Material to the Article:

**“The role of social deprivation and depression in dementia risk: Findings from the longitudinal Survey of Health, Ageing and Retirement in Europe”**

**Table S1. Chronic conditions asked about in the Survey of Health, Ageing and Retirement in Europe (SHARE)**

| Question | Answer options |
| --- | --- |
| “Has a doctor ever told you that you had/ Do you currently have any of the conditions on this card? “ | 1) A heart attack including myocardial infarction or coronary thrombosis or any other heart problem including congestive heart failure |
|  | 2) High blood pressure or hypertension |
|  | 3) High blood cholesterol |
|  | 4) A stroke or cerebral vascular disease |
|  | 5) Diabetes or high blood sugar |
|  | 6) Chronic lung disease such as chronic bronchitis or emphysema |
|  | 7) Asthma |
|  | 8) Arthritis, including osteoarthritis, or rheumatism |
|  | 9) Osteoporosis |
|  | 10) Cancer or malignant tumour, including leukaemia or lymphoma, but excluding minor skin cancers |
|  | 11) Stomach or duodenal ulcer, peptic ulcer |
|  | 12) Parkinson disease |
|  | 13) Cataracts |
|  | 14) Hip fracture or femoral fracture |
|  | 15) Other fractures |
|  | 16) Alzheimer’s disease, dementia, organic brain syndrome, senility or any other serious memory impairment |
|  | 17) Benign tumor (fibroma, polypus, angioma) |

**Table S2. Demographics by self-reported dementia diagnosis status.**

|  | Self-Reported Dementia Diagnosis | No Self-Reported Dementia Diagnosis | *P*-Value* |
| --- | --- | --- | --- |
| N= | 509 | 11, 114 |  |
| Gender |  |  | .317 |
| *Male* | 232 (45.58 %) | 5329 (47.95 %) |  |
| *Female* | 277 (54.42 %) | 5785 (52.05 %) |  |
| Age |  |  | <.001 |
| *Mean (SD)* | 73.65 (8.22) | 64.46 (8.43) |  |
| *Range* | 50 – 97 | 50 – 97 |  |
| Yrs Education |  |  | <.001 |
| *Mean (SD)* | 9.12 (4.77) | 11.00 (4.42) |  |
| *Range* | 0 – 25 | 0 – 25 |  |
| Marriage Status |  |  | <.001 |
| *Married/Partnered* | 344 (67.58 %) | 8, 435 (75.90 %) |  |
| *Widowed/Divorced/*  *Never Married* | 165 (32.42 %) | 2, 679 (24.10 %) |  |
| Chronic Conditions |  |  | <.001 |
| *Mean (SD)* | 1.92 (1.54) | 1.41 (1.34) |  |
| *Range* |  |  |  |
| Depressive Symptom Status |  |  | <.001 |
| *No past/ baseline* | 382 (75.05%) | 9,465 (85.16%) |  |
| *Past symptoms* | 49 (9.63%) | 923 (8.31%) |  |
| *Present symptoms* | 36 (7.07%) | 374 (3.37%) |  |
| *Stable symptoms* | 42 (8.25%) | 352 (3.17%) |  |
| Income |  |  | <.001 |
| *Median (IQR)* | 18, 400 (17, 648) | 23, 307 (26, 099) |  |
| *Range* | 0 – 367, 735 | 0 – 1, 218, 168 |  |
| Wealth |  |  |  |
| *Median (IQR)* | 7, 000 (39, 964) | 16, 000 (66, 278) | <.001 |
| *Range* | -191, 240 – 900, 000 | -505, 782 – 1, 789,786 |  |
| Health Insurance Status |  |  | .002 |
| *Basic* | 323 | 6, 272 |  |
| *Supplementary* | 186 | 4, 842 |  |
| Ever held a job for 5+ years |  |  | .690 |
| *Yes* | 489 (96.07 %) | 10, 726 (96.51 %) |  |
| *No* | 20 (3.93 %) | 388 (3.49 %) |  |

* Difference in Age calculated using a t-test. All other continuous variables calculated with Mann-Whitney U Test, categorical variables calculated with Chi-Square-Tests.

Abbreviations: IQR, interquartile range; N, total number; NA, not applicable; SD, standard deviations, Yrs, years.

**Table S3. Fine-Grey regression results for the full sample.**

|  | Model without Depressive Symptom Status | | | Model with Depressive Symptom Status | | |
| --- | --- | --- | --- | --- | --- | --- |
| Variable | **HR**  **(95% CI)** | ***P-*Value** | **SE** | **HR**  **(95% CI)** | ***P-*Value** | **SE** |
| SDI |  |  |  |  |  |  |
| Low | REF |  |  | REF |  |  |
| Moderate | 1.27 (0.95-1.69) | *.100* | 0.18 | 1.25 (0.94-1.67) | *.120* | 0.18 |
| High | 1.78 (1.31-2.43) | ***<.001*** | 0.28 | 1.64 (1.20-2.24) | ***.002*** | 0.26 |
| Depressive Symptom Status |  |  |  |  |  |  |
| No Past/Baseline Depressive Symptoms |  |  |  | REF |  |  |
| Past Depressive Symptoms |  |  |  | 1.61 (1.19-2.17) | ***<.001*** | 0.25 |
| Baseline Depressive Symptoms |  |  |  | 1.47 (1.04-2.06) | ***.027*** | 0.26 |
| Stable Depressive Symptoms |  |  |  | 2.85 (2.04- 3.99) | ***<.001*** | 0.49 |
| Gender |  |  |  |  |  |  |
| Female | REF |  |  | REF |  |  |
| Male | 0.74 (0.62-0.89) | ***.002*** | 0.07 | 0.80 (0.66-0.97) | ***.020*** | 0.08 |
| Marriage Status |  |  |  |  |  |  |
| Married/Partnered | REF |  |  | REF |  |  |
| Not Married/Partnered | 0.72 (0.60-0.88) | ***.001*** | 0.07 | 0.72 (0.60-0.88) | ***<.001*** | 0.07 |
| Chronic Conditions | 1.00 (0.94-1.06) | *.965* | 0.03 | 0.96 (0.90-1.02) | *.210* | 0.03 |
| Wald-test | 34.53 | | | 81.79 | | |
| *P-*Value | *<.001* | | | *<.001* | | |
| df | 5 | | | 8 | | |
| BIC | 8075 | | | 8056 | | |

Abbreviations: BIC, Bayesian information criterion; CI, confidence interval; df, degrees of freedom; HR, hazard ratio; REF, indicates the reference level of a variable; SE, standard error.

**Supplementary Text S1. Sensitivity analysis using cognitive testing performance as the outcome.**

**Methods**

**Outcome measure**

Cognitive testing in the SHARE assessed word recall, orientation, numeracy, and verbal fluency. Respondents were asked to reproduce a list of 10 words, both immediately and after a delay (approx. 5 minutes). Each correctly recalled word was worth one point at each instance (range: 0–20). For a measure of their orientation, respondents were asked about the current date (scored 0–4, one point each for correct day of the month, month, year, and day of the week). Numeracy was assessed using four questions on percentages (scored 0–4, one point for each correct answer). For the verbal fluency test, respondents list the names of as many animals as they can think of in 1 minute (range: 0–100). We calculated age-adjusted Z-scores of performance in each domain and took the average of these scores to achieve a global cognitive Z-score.

**Analysis**

Those who at baseline showed a performance indicative of cognitive impairment (<=10% percentile; see e.g. Hayat et al., 2014; Thomann et al., 2020) were excluded. This resulted in a subsample of n=10,460. Within this subsample, main analysis were repeated to estimate risk of cognitive impairment (i.e. performance dropping <=10% percentile).

**Results**

Both moderate (HR= 1.90 [95% CI: 1.47-2.46], P<.001) and high (HR= 2.84 [95% CI: 2.17-3.71], P<.001) SoDep Index Status were associated with an increased risk of objective cognitive impairment, compared to low SoDep Index Status. Adding Depressive Symptom Status as a predictor, the hazard ratios did not change for the moderate SoDep Index Status, while changing slightly for the high SoDep Index Status (HR= 2.81, [95% CI: 2.15-3.67], P<.001). This suggests that SoDep Index Status’ direct association with risk of cognitive impairment explained the majority of variance.

**Figure S1. Kaplan-Maier curves indicating lack of feasibility of Depressive Symptom Status by SoDep Index Status interaction analysis.**

**
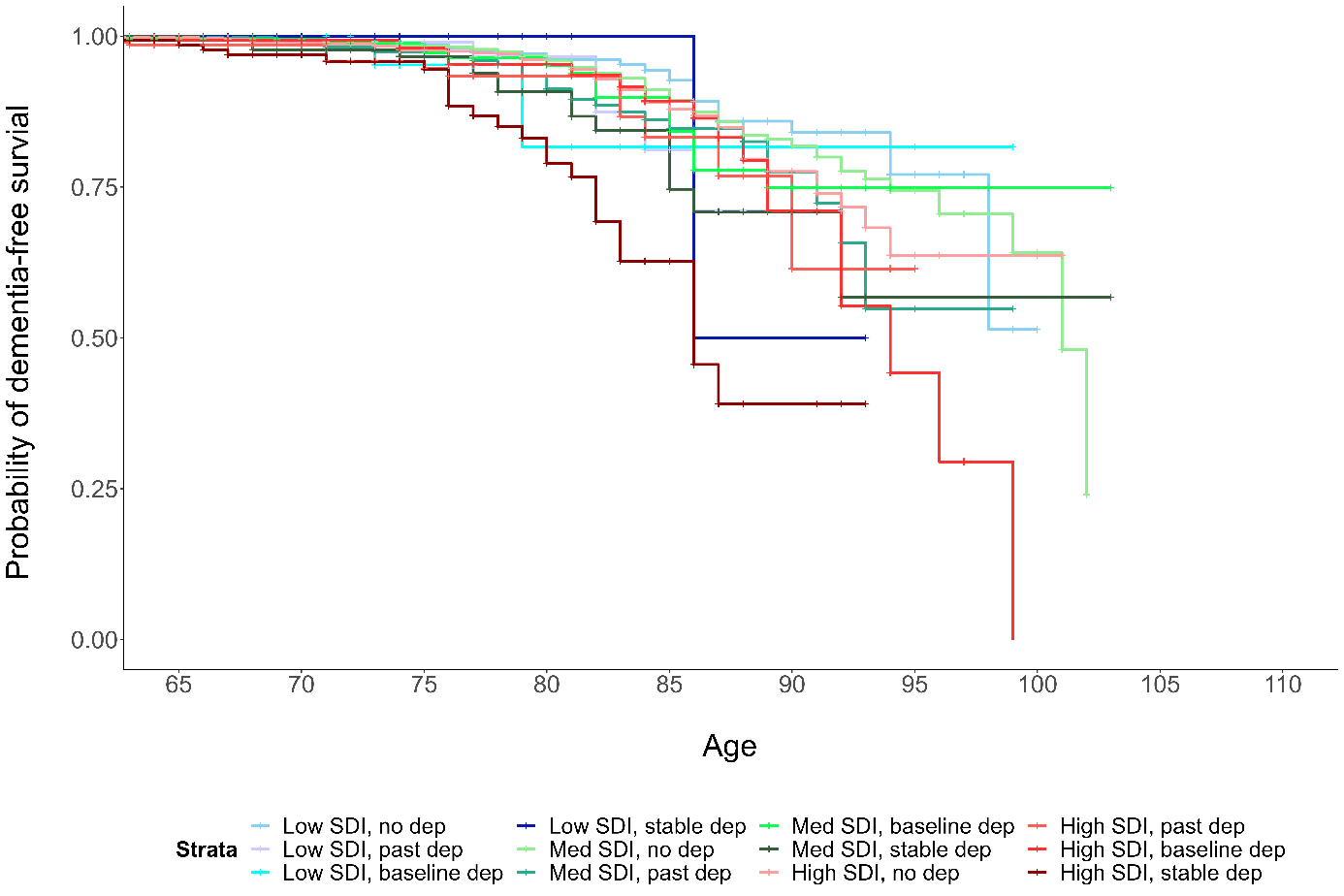
**

Abbreviations: dep = Depressive Symptom Status; SDI= SoDep Index Status
